# Experiences With a Self-Reported Mobile Phone-Based System Among Patients With Colorectal Cancer: A Qualitative Study

**DOI:** 10.2196/mhealth.5426

**Published:** 2016-06-09

**Authors:** Jenny Drott, Maria Vilhelmsson, Karin Kjellgren, Carina Berterö

**Affiliations:** ^1^ Division of Nursing Science Department of Medical and Health Sciences Linköping University Linköping Sweden; ^2^ Division of Nursing Science Department of Medical and Health Sciences University of Linköping Linköping Sweden

**Keywords:** cancer, conventional content analysis, informatics technology systems, mHealth, self-reported mobile phone-based system, symptom monitoring

## Abstract

**Background:**

In cancer care, mobile phone-based systems are becoming more widely used in the assessment, monitoring, and management of side effects.

**Objective:**

To explore the experiences of patients with colorectal cancer on using a mobile phone-based system for reporting neurotoxic side effects.

**Methods:**

Eleven patients were interviewed (ages 44-68 years). A semistructured interview guide was used to perform telephone interviews. The interviews were transcribed verbatim and analyzed with qualitative content analysis.

**Results:**

The patients' experiences of using a mobile phone-based system were identified and constructed as: “being involved,” “pacing oneself,” and “managing the questions.” “Being involved” refers to their individual feelings. Patients were participating in their own care by being observant of the side effects they were experiencing. They were aware that the answers they gave were monitored in real time and taken into account by health care professionals when planning further treatment. “Pacing oneself” describes how the patients can have an impact on the time and place they choose to answer the questions. Answering the questionnaire was easy, and despite the substantial number of questions, it was quickly completed. “Managing the questions” pointed out that the patients needed to be observant because of the construction of the questions. They could not routinely answer all the questions. Patients understood that side effects can vary during the cycles of treatment and need to be assessed repeatedly during treatment.

**Conclusions:**

This mobile phone-based system reinforced the patients’ feeling of involvement in their own care. The patients were comfortable with the technology and appreciated that the system was not time consuming.

## Introduction

The number of mobile subscriptions worldwide is estimated to be almost 7 billion. The growth rate until 2014 reached 2.6% globally, which is a low level indicating that the market is approaching saturation levels [1]. The continuous increase in mobile subscriptions is mostly due to growth in the developing world. The Global Observatory for eHealth of the World Health Organization defines mobile health (mHealth) as a “medical and public health practice supported by mobile devices such as mobile phones, patient monitoring devices, personal digital assistants, and other wireless devices.” The mobile phones of today have capacious memories, large screens, and operating systems that encourage the development of applications and other mobile phone-based systems [2]. There are great opportunities for mHealth in using these technologies.

The type of technology used in this study, mHealth, has been used by health care providers in home care for symptom management of patients with chronic diseases. Mobile phones are used for patients with chronic diseases such as hypertension, diabetes, heart disease, and asthma and also for a range of other health problems. Apart from enhancing the capacity to self-manage long-term conditions, mobile phone technology can have an impact on the understanding of a disease. It can also assist in lifestyle modifications and creates a supportive environment [3]. This may in turn have an effect on patients' independence, responsibility, and self-esteem. Mobile phone technology has advantages for users when it comes to portability, immediacy, convenience, comparatively low cost, efficiency, and simple usability [4]. Mobile phone-based health systems facilitate communication between patients and health care providers [3,4]. The increased number of mobile phone users creates new possibilities in cancer care.

Treatment of patients with advanced colorectal cancer (CRC) involves a postoperative combination of different chemotherapeutic agents. Oxaliplatin is one of the drugs used today, usually combined with 5-fluorouracil (5-FU) and folinic acid (leucovorin) [5,6]. Oxaliplatin is known to be highly neurotoxic, and neurotoxic side effects occur in most patients [7]. Severe neurotoxic side effects can affect the patient’s daily life, limiting physical functions, and can be associated with depression and affected quality of life [8]. It is of great importance to identify patients who are at risk of developing high-grade neurotoxicity [6]. Early identification can eliminate the risk of developing chronic neurotoxic side effects with functional impairment [9]. Health care professionals have an important role in supporting patients and identifying and reporting early signs of neurotoxicity. It is necessary to have valid questionnaires to capture neurotoxic signs during and after oxaliplatin treatment [10,11].

The increased number of mobile phone users creates new possibilities for interventions with mobile phone-based technology. Such technology is useful for cancer care in prevention and early detection of cancer, treatment follow-up, and patient-health professional communication [12]. Mobile phone-based systems related to cancer care seem to have limited use and are employed only during limited phases of the care process. There is a need to improve the reliability and quality of content of what to be assessed by involving the medical profession in designing the systems [13,14]. In previous studies, patients used mobile phone-based systems to answer symptom questionnaires during treatment and to receive advice via the mobile phone [15,16]. Patients using mobile phone-based systems experienced improved management of their side effects. They felt secure knowing that their side effects were monitored by their health care provider and that they were participating in their own care [16,17]. In cancer care, mobile phone-based systems are becoming more widely used in the assessment, monitoring and management of side effects, self-care, and advice [17-19]. Few studies have used a mobile phone-based reporting system to monitor chemotherapy-induced side effects and to visualize distress with graphs and act on it. There are also few studies evaluating the usability of mobile phone-based systems in cancer care [18,19].

### CQ: A Mobile Phone-Based System

In this study, a mobile phone-based system for self-reporting was used. This solution allows patients to answer structured questionnaires on their own mobile phones regarding their health and side effects of treatment. Patients’ neurotoxic side effects in this study were identified in a self-reported, mobile phone-based system named Circadian Questions (CQ) (21st Century Mobile AB, http:// www.cqmobil.se). The CQ mobile phone-based system is designed to be platform independent. The CQ system is compatible with JAVA ME phones, iPhone, Android, iPad, and Windows Phone. The patients received platform-specific written information regarding installation of the system on their mobile phones.

**Figure 1:**
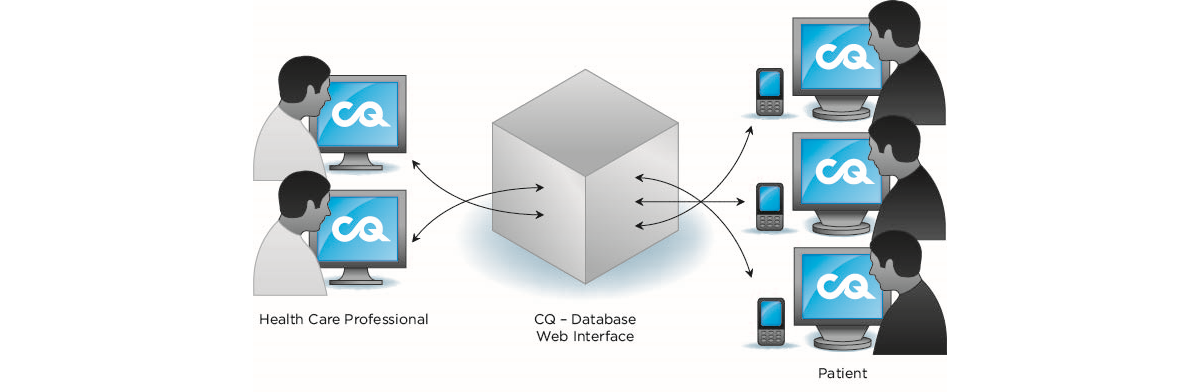
The collected and identified data are transferred safely to a secure database via the Internet as data traffic (reproduced with permission from 21st Century Mobile AB).

The collected, identified data are transferred to a secure database via the Internet as data traffic, not SMS (Short Message Service) (Figure 1). 

The answers are made available in real time and presented as graphs to the authorized health care professionals after they log in to a web interface. The cost for the patients is very low thanks to the use of data traffic; a set of answers (4K) costs 0.5 € cents as a maximum and the installation SMS at the start of the study costs roughly 10-20€ cents.

**Figure 2:**
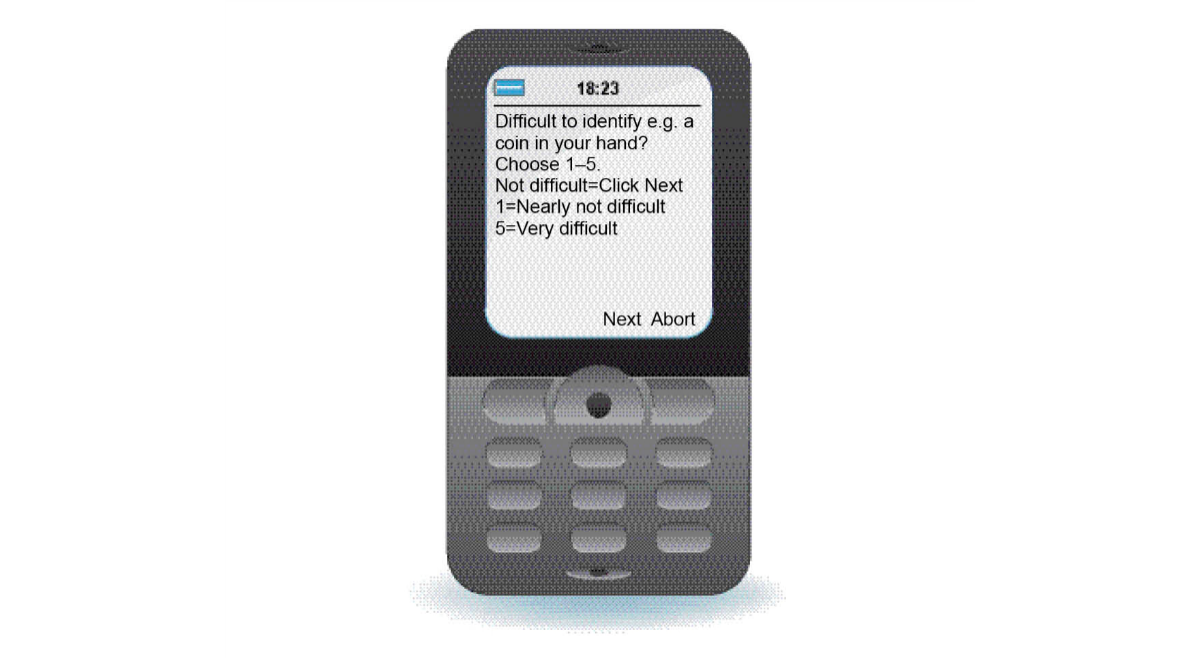
Example of a condensed question in the mobile phone display (reproduced with permission from 21st Century Mobile AB).

The research group modified and adjusted the Swedish version of the questionnaire OANQ to fit all mobile phone displays that are compatible with CQ [10]. In the adjustment process, some of the questions were condensed and some sentences shortened so that they would fit even the smallest displays of the JAVA ME mobile phones (many patients still use JAVA mobile phones), but this did not interfere with the content of the questionnaire. Example: *difficulty identifying objects in your hand (eg, coin)* was condensed to; *difficulty identifying (eg, a coin in your hand)* (Figure 2). All the questions had a similar structure, and the patients answered them in a numerical rating scale in the mobile phone by pressing a number between 1 and 5. The condensed questions were tested on some of the patients’ representatives in advance to check that the content and understanding of the questions were preserved.

When the health care professionals initiated the transfer of the questionnaire to the individual mobile phone, they used a calendar function to fill in the specific dates that the patient had received the questions. The dates in the calendar were set according to each patient's specific chemotherapy regime. The calendar was adapted to each specific patient, and the chosen questionnaire was sent out to the patient at exactly the right moment in the treatment cycle to attain individual customized assessments. This calendar function was specifically developed for this project or study to enhance individual customized measurements.

The aim of this study was to explore the experiences of patients with CRC of using a mobile phone-based system for reporting neurotoxic side effects.

## Methods

### Study Population

This study is a part of a larger study with descriptive data of the frequencies of existing neurotoxicity and impact on daily life in patients with CRC cancer. Eleven patients were included in the present study from March to August 2014. One patient declined participation because of serious illness and a long hospital stay. Patients were included from 4 hospitals in the south of Sweden—2 university hospitals and 2 regional hospitals. Patient’s inclusion criteria were as follows: at least 18 years of age; had been treated with 5-fluorouracil (5-FU) and folinic acid (leucovorin) in combination with oxaliplatin postoperatively in an adjuvant setting for stage II-III CRC; ability to speak and understand the Swedish language; and had answered CQ questionnaires on their own mobile phone to assess neurotoxicity. Every patient answered questionnaires using the mobile phone-based system CQ. At the time of the telephone interview, the patients had diverse experiences of answering the questionnaires using their mobile phones. The time they had used CQ varied from 1 to 10 months.

### Data Collection

Data were collected through qualitative interviews. One of the authors (MV) contacted the patients and interviewed them by telephone. A semistructured interview guide was used. The questions asked were (1) what did you think of answering the questionnaire with repeated questions via a mobile phone? (2) what did you think of registering your side effects via a mobile phone? (3) were there any advantages in answering questions via a mobile phone? (4) where there any disadvantages in answering questions via a mobile phone? (5) could something in the mobile phone reporting system be improved? To clarify some parts of the interview, questions such as, “can you tell me more about that?” or “can you clarify that?” were asked. The interviews were audio digitally recorded and lasted between 12-31 minutes (mean 20). The interviews were transcribed verbatim.

### Analysis

With the explorative aim of the study in mind, the respondents’ transcribed interviews were analyzed as a whole using techniques of conventional qualitative content analysis [20,21]. Qualitative content analysis can be applied to transcribed interviews because there is a text to work with. The analyses were performed by three of the authors (JD, MV, and CB). Before beginning the analyses, the authors read the transcribed interviews. Statements with similarities were grouped together and summarized into tentative clusters based on the questions asked. These tentative clusters were reviewed in detail, and all the included statements were scrutinized. Unclear statements were explored with respect to the original context. Through iterative in-depth discussions among all the authors, the statements were reclustered step by step, and a more logical and complete structure gradually emerged. These completed structures of original text were again reviewed in their original context and condensed into final clusters by the three authors, and final adjustments were made. Thus, all the clusters were validated through systematic repeated reviews of the data. To confirm and illustrate the clusters identified, quotations related to the clusters were selected during the analysis process. The quotes used in the Results section illustrate examples of patients’ statements. The interviews and transcriptions were all in Swedish. To produce this paper, great care was taken to translate the quotations with the support of a native, English-speaking translator.

### Ethics

All data were treated carefully and confidentially. All patients provided oral and written informed consent in line with the Declaration of Helsinki [22]. Ethical approval was obtained from the Regional Ethical Review Board (record no.: 2012/301-31).

## Results

Eleven patients were included in the analysis, 4 men and 7 women. They were aged between 44-68 years, median 65.

Three main clusters were identified and constructed during the analysis, and given the names “being involved,” “pacing oneself,” and “managing the questions.” These are shown in Table 1. Each of the main clusters includes a number of subclusters at varying levels of abstraction, and the clusters are linked together by the underlying meanings.

**Table 1:** Overview of clusters and subclusters.

Being involved	Pacing oneself	Managing the questions
Being a participant	Having impact	Need to be alert
Being aware	Having support	Recognition
Getting knowledge		Discovering “the gray area”
Being a contributor		

### Being Involved

The patients experienced that they were *participating* in their own care in various ways when they used the self-reporting mobile phone-based system. Patients were aware that the answers they gave were monitored in real time and taken into account by health care professionals when planning further treatment.

...they were very careful, at least when I had the treatment, to check up on these questions. They’ve changed my medication several times and now I’m doing really well. So that’s very positive.

Patient no.: 8

...it’s very good if someone can do a follow-up immediately after you’ve sent the answers and that you get, as in my case, a question or someone giving you a call who says that you’ve answered this and that and we’d like to talk to you about it and so on. That was great. I think that’s very useful, definitely.

Patient no.: 3

This reporting system was perceived to be flexible and patients were comfortable with it. The results of the study point out the usability of the system. The initial reaction to the questionnaires was that they contained a large number of questions, but patients found them relevant to the treatment and therefore meaningful. If the patients had a problem such as a hearing disability, it was more convenient to answer questions via the questionnaire on the mobile phone than having a conversation on the telephone. Patients stated that answering a questionnaire on the mobile phone was easier and less time consuming than answering a paper questionnaire and having to post it. Patients reflected on the different ways of communicating and personal contact with medical staff.

I think it can be a really good instrument // I don't think that you can ignore the importance of a physical meeting completely. You might think it’s enough to answer the questionnaire, you have an alternative.

Patient no.: 2

Answering the questionnaires also made the patients more *aware* of the side effects, both in a positive way and in a negative way. This awareness provided the patients and the health care professionals with *knowledge* about the side effects of treatment. Patients were faced with aspects of the diagnosis that could be a strain emotionally, and sometimes upsetting. This is something that can be found in all forms of questionnaire, as they can make patients aware of consequences and effects they have not thought about before.

The patients had a sense of *contributing* to something more beside their own care. By answering the questionnaires via their mobile phones, they contributed to extending and developing oncology care and/or nursing.

…with my participation I am a part of development of oncological treatment. It is about understanding how patients are doing during treatment and also about decreasing side effects for people that need to have this chemotherapy in the future.

Patient no.: 6

### Pacing Oneself

The main reason for the patients' appreciation of this self-reporting system was the possibility to *pace oneself*. As a patient, you have options regarding when and where you want to answer the questionnaire; you can choose a time and a place to answer the questions and you can do it in peace and quiet. For patients, this was an important advantage of using a mobile phone-based system. A questionnaire on paper would probably have resulted in patients' withdrawal from the study. A reminder to answer the questionnaire was sent out to the patient’s mobile phone at 4 pm if the questionnaire had not been answered before that time.

The reminder is good, since it’s not always suitable to answer the questionnaires at the first “mailing.” // Questionnaires on the mobile phone could be answered almost anywhere and whenever you wanted to.

Patient no.: 3

I can answer when I want. For example, the last time I answered the questions I was sitting in my car on the way home from Stockholm. Well, the accessibility… and I can decide for myself. // More than if we were to have telephone contact. This is freedom, I can do it wherever I want to.

Patient no.: 7

Answering the questions via this self-reporting system was perceived as easy and not time consuming. The process of answering was quickly completed considering the total number of questions. The questions in the original questionnaires were quite long, so when using them in the mobile phone-based system, the questions were shortened. The patients stated that the questions were condensed, but were clear and easy to answer.

It’s only a few “clicks” and it’s very easy. Especially when you just answer with a number and don’t have to write things down.

Patient no.: 4

I always have my mobile phone with me, so it’s easier. It’s quick and better than surveys on paper and computers.

Patient no.: 5

The system was not difficult for the patients when it comes to usability. However, some patients encountered technical problems or needed help with the functioning of the questionnaire. They all had opinions about the *support*; support was just a phone call away and easily accessible. On some occasions, there were glitches in the system due to problems with the mobile network accessibility in the countryside.

### Managing the Questions

The patients *needed to be alert*, at least at the beginning of the study, when answering the questions. Patients pointed out that they could not routinely answer the questions in the beginning. They needed to be observant due to the construction of the questions.

You need to observe how the questions are formulated. The construction of the questions sometimes differs, which affects how you value the answer on the scale. The questionnaire demands that you are alert and really read what is stated in each question.

Patient no.: 1

I think there are a lot of questions but the questions are similar, so I learned to manage them.

Patient no.: 9

After a while, there was *recognition*; there were a great number of questions, but the patients found that acceptable because they became familiar with the structure. Even though there were many questions, the patients stated that they managed to answer them smoothly and quickly.

At the beginning, some of the patients had trouble understanding why the questions were repeated continuously at certain intervals. Gradually there was an understanding of the need for this repetition because the side effects could vary during treatment. Patients recognized the fact that the same side effects needed to be measured before and after every dose of chemotherapy to identify and follow-up the side effects of treatment.

The treatment I receive affects my entire body, so I don't think that it's possible to reduce the number of questions. These are the side effects I usually have, so, well I don’t think that it’;s possible to reduce the number of questions.

Patient no.: 4

Some patients pointed out that they had side effects that could not be conveyed in the questionnaire, and therefore asked for space to write comments. The patients highlighted so called “gray areas” when trying to answer the questions; there were side effects that could not be defined by a number on the scale and there were problems that patients wished to explain using words instead. When patients thought they were in this “gray area,” they would have preferred to talk to a physical person and explain their issues.

… but it’s a bit difficult, you can’t give details, you have to give a number. For example, if you have problems with eyesight. You know, I press on one eye, left eye, and the eyesight disappears completely. Then it comes back gradually when I release the pressure. So that’s a side effect I can’t get across with the questions.

Patient no.: 11

There are questions that I cannot identify with, and sometimes it can be hard to select the proper rating for my side effects.

Patient no.: 10

Patients could have distressing and painful side effects such as pricking sensations but they were able to cope with them. Some patients received phone calls from health care personnel who were observing the answers. Patients were grateful for the telephone contact and considered this as a bonus in the study.

## Discussion

The findings of this study highlight the experiences of patients with CRC of using a mobile phone-based system for reporting neurotoxic side effects. Patients felt involved in their own care. Symptom monitoring by means of a mobile phone gave the patient the opportunity to communicate the neurotoxic side effects of the treatment to medical staff, who could act on it within a short period. Real-time symptom monitoring gives an accurate image of the patient’s experience of the side effects. Adaption of individual doses of chemotherapy is possible using mobile phone technology according to Weaver et al [16]. Real-time monitoring of toxicity enables optimization of dose increase and effective management of side effects [16]. As reported in another study, delayed self-reporting of chemotherapy side effects can lead to weaker insights into the symptom burden, partly due to patients forgetting the severity of the side effects [23].

There was an increased awareness of neurotoxic side effects among patients. Identification of neurotoxic side effects in patients with CRC can eliminate the risk of functional impairment regarding chronic neurotoxicity [9]. The mobile phone-based system used in this study is constructed for individual flexibility and enables the questions to follow every patient’s specific cancer treatment regime.

According to De Jongh et al [24], mobile phone-based systems may facilitate self-management of long-term illnesses. Patients express interest in using these kinds of mobile phone-based system. However, the evidence for this is based on a small number of trials, and further research is needed to gain more information about the long-term effects, acceptability, costs, and risks of such interventions. The mechanisms behind short- and long-term acceptability, such as message content and frequency, need further research [24].

The issues of time spent on answering the questionnaire and accessibility of mobile phone-based system were essential for patients. The patients appreciated that the system was not time consuming. The questions in the questionnaires were condensed to fit the displays of all mobile phones compatible with the CQ system [11]. Patients found them easy to understand and meaningful to answer. The CQ system is not designed for a particular mobile phone platform. It can thus be used on a wide range of different platforms, which makes it accessible to a large proportion of the population.

It is necessary, though, that the side effects are followed by the medical staff and the health care professionals by means of graphs. In a study by McCann et al [17], they used an automated system where health care professionals observed patients' side effects of chemotherapy-related toxicity in real time. Patients reported symptoms on days 1-14 after their first 4 cycles of chemotherapy. Data were registered in a system, and advice was sent to the patient [17].

Today, short hospital stays are common, and many patients are required to recover at home. For some patients, this can be convenient and improve overall quality of life. But again, other patients may need prolonged support and medical assistance to be able to recover after their hospital stay. Monitoring these patients and conducting follow-up visits are very time consuming for health care professionals. This is problematic given the increasing workload for health care staff today. A system that facilitates the contact between the patients and the oncology team with early detection of side effects in chemotherapy treatment could not only improve patients’ health but also be cost-efficient. In a study about hypertension management, health care professionals stressed the importance of being accessible to patients and that an interactive self-report system increased the contact with patients. In that study, patients’ self-management of their hypertension was examined [25,26]. Feedback provided by health care staff is appreciated by patients as a “bonus” and a confirmation of the fact that they could have an impact on their own care. The prevention of future complications and improved cancer treatment are of great importance to the patients. Mobile phones have previously been successfully used in different areas of health care. Even so, there are surprisingly few research studies that focus on mobile phone technology for disease and health monitoring [27,28].

In the larger study by Drott et al [29], neurotoxic side effects were monitored for a considerably longer period of time than in previous studies. Previous studies have followed patients for a few cycles of treatment [15,16]. Our study is unique in that the patients’ neurotoxic side effects were registered in CQ from the start of chemotherapy and up to 1 year after treatment. Every patient answered the CQ questions 4 times during every treatment cycle. In this study, the 11 individuals were at different stages in their chemotherapy treatment and therefore they had answered questionnaires in the CQ system for varying lengths of time. The interval at which questionnaires were sent out could be adjusted individually to each specific patient thanks to the calendar function. Real-time symptom monitoring and longitudinal follow-up of side effects give a proper impression of the patient’s experience and symptom burden.

In cancer care, mobile phones are becoming more widely used in the assessment, monitoring and management of side effects, self-care, and advice [17-19]. Our results are unique because our mobile phone-based system is individually tailored for each patient. We also focus on neurotoxicity, and there are no other studies that measure neurotoxicity in real time, so frequently and for such a long time. Some patients pointed out that they had side effects that could not be conveyed in the questionnaire, and therefore asked for space to write comments. The patients highlighted so-called “ *gray areas”* when trying to answer the questions; there were side effects that could not be defined by a number on the scale and there were problems that patients wished to explain using words instead. When the patients perceived themselves as in this “ *gray area*,” they would have preferred talking to a physical person, to explain their issues. These results pointed out that mobile phone-based system is a complement to the physical meeting with medical staff.

### Limitations of the Study

This study has some limitations. The sample size of 11 participants may seem small, but this sample size is relevant in qualitative research [30]. This is a starting point for further research and with respect to the diagnosis of cancer, researchers have to take into account vulnerable groups of patients, makes it difficult to find patients eligible for studies. The telephone interviews in this study were in addition to the regular care routine. Telephone interviews are generally shorter than face-to-face interviews, and nonverbal information is lost. An advantage of telephone interviews may be that they save time because no one involved in the interview needs to travel to a physical meeting. As in all interviews and surveys, there could be a risk of including only those people with a positive attitude.

### Conclusions

The results of this study show that this mobile phone-based system reinforced the patients’ feeling of involvement in their own care. The patients felt comfortable with this mobile phone-based technology, it was accessible and usable.

## Abbreviations

CQ: Circadian Questions
CRC: Colorectal Cancer
mHealth: mobile health
